# Supplementary material for: Association between predialysis creatinine and mortality in acute kidney injury patients requiring dialysis
Source: PLoS One. 2022 Sep 26;17(9):e0274883. doi: 10.1371/journal.pone.0274883 (PMC9512211; doi:10.1371/journal.pone.0274883)
Supplement: S1 Table — (DOCX) [file pone.0274883.s001.docx]

**Supplement Table 1**. ICD-9 diagnosis codes used to identify acute kidney injury, transplant history, and comorbidities.

| Diagnosis | ICD-9 diagnosis codes |
| --- | --- |
| Acute kidney injury | 584.x, 634.3, 635.3, 636.3, 637.3, 638.3, 639.3, 669.3, 958.5 |
| Diabetes Mellitus (DM) | 249,250 |
| Hypertension (HTN), | 401,402,403,404,405 |
| Chronic Kidney Disease (CKD) | 585,586 |
| Liver Cirrhosis (LC) | 571.2,571.5,571.6,572.2,572.3,572.4,572.8 |
| Cancer | between 196.0 and 199.1, between 209.70 and 209.75, 209.79, 789.51 / Metastatic cancer / |
|  | between 200.00 and 202.38, between 202.50 and 203.01, between 203.02 and 203.82, 238.6, 273.3 / Lymphoma / |
|  | between 140.0 and 172.9, between 174.0 , between 258.01 and 258.03, between 209.00 and 209.24, between 209.25 and 209.30, 175.9, 179, 195.8, 209.30, 209.36  / Solid tumor without metastasis / |
| CKD stage 4 and 5 | 585.4, 585.5 |
| End-stage kidney disease | 585.6 |
| Transplant | V42, 996.8 |
