# Supplementary material for: Association between predialysis creatinine and mortality in acute kidney injury patients requiring dialysis
Source: PLoS One. 2022 Sep 26;17(9):e0274883. doi: 10.1371/journal.pone.0274883 (PMC9512211; doi:10.1371/journal.pone.0274883)
Supplement: S2 Table — (DOCX) [file pone.0274883.s002.docx]

**Supplement Table 2.** Drug names of diuretics and vasopressors.

| Drug type | Drug name |
| --- | --- |
| Diuretics | Bumetanide, Bumex, Furosemide, Lasix |
| Vasopressors | Norepinephrine, Epinephrine, Vasopressin, Dopamine, Dobutamine, Phenylephrine |
