# Supplementary material for: Association between predialysis creatinine and mortality in acute kidney injury patients requiring dialysis
Source: PLoS One. 2022 Sep 26;17(9):e0274883. doi: 10.1371/journal.pone.0274883 (PMC9512211; doi:10.1371/journal.pone.0274883)
Supplement: S3 Table — (DOCX) [file pone.0274883.s003.docx]

**Supplement Table 3.** Percentages of missing data.

| Database | eICU | MIMIC |
| --- | --- | --- |
| BUN (mg/dL) | 0.29% | 0% |
| FiO2 (%) | 22.73% | 25.43% |
| O_2_ Sat (%) | 2.63% | 30.92 |
| HCO_3_ (mmol/L) | 2.48% | 0.18% |
| Hgb (mg/dL) | 4.07% | 0.82% |
| WBC count (×1000/μL) | 4.96% | 0.64% |
| Albumin (g/dL) | 25.35% | 51.23% |
| Anion gap (mmol/L) | 20.59% | 1.18% |
| Calcium (mg/dL) | 2.13% | 6.31% |
| Chloride (mmol/L) | 0.25% | 0% |
| Creatinine (mg/dL) | 0% | 0% |
| Glucose (mg/dL) | 0.09% | 0.09% |
| Platelet count (×1000/μL) | 4.91% | 0.36% |
| Potassium (mmol/L) | 0.19% | 0% |
| Sodium (mmol/L) | 0.09% | 0% |

*Abbreviations*: BUN, blood urea nitrogen; FiO_2_, fraction of inspired oxygen; Hgb, hemoglobin; WBC, white blood cell.
