# Supplementary material for: Association between predialysis creatinine and mortality in acute kidney injury patients requiring dialysis
Source: PLoS One. 2022 Sep 26;17(9):e0274883. doi: 10.1371/journal.pone.0274883 (PMC9512211; doi:10.1371/journal.pone.0274883)
Supplement: S4 Table — (DOCX) [file pone.0274883.s004.docx]

**Supplement Table 4.** Comparison of variables between the eICU and MIMIC datasets.

| Variables | eICU | MIMIC | *P* value |
| --- | --- | --- | --- |
| Demographics |  |  |  |
| Sex, % men | 940 (58.8%) | 432 (62.2%) | 0.128 |
| Age, years | 62.6±14.5 | 62.9±16.1 | 0.588 |
| Black race, % | 199 (12.4%) | 67 (9.7%) | <0.001 |
| Comorbidity, % |  |  |  |
| Diabetes mellitus | 180 (11.2%) | 224 (32.3%) | <0.001 |
| Hypertension | 179 (11.2%) | 348 (50.1%) | <0.001 |
| CKD | 206 (12.9%) | 123 (17.7%) | 0.003 |
| Malignancy | 76 (4.8%) | 67 (9.7%) | <0.001 |
| Liver cirrhosis | 102 (6.4%) | 127 (18.3%) | <0.001 |
| Medication, % |  |  |  |
| Diuretics | 197 (12.3%) | 156 (22.5%) | <0.001 |
| Vasopressors | 699 (43.7%) | 248 (35.7%) | <0.001 |
| Laboratory data |  |  |  |
| BUN (mg/dL) | 62.1±37.2 | 65.9±38.3 | 0.025 |
| FiO_2_ (%) | 51.3±25.9 | 47.9±26.1 | 0.004 |
| Hgb (mg/dL) | 9.7±2.1 | 10.1±1.5 | <0.001 |
| O_2_ Sat (%) | 95.1±5.8 | 90.3±10.8 | <0.001 |
| WBC count (×10^3^/μL) | 16.0±24.1 | 16.2±31.0 | 0.881 |
| Albumin (g/dL) | 2.7±0.7 | 2.8±0.8 | <0.001 |
| HCO_3_ (mmol/L) | 20.5±5.7 | 20.1±5.0 | 0.135 |
| Anion gap (mmol/L) | 14.6±6.3 | 19.7±5.3 | <0.001 |
| Calcium (mg/dL) | 8.0±1.0 | 8.4±1.2 | <0.001 |
| Creatinine (mg/dL) | 4.4±3.2 | 4.1±2.6 | 0.017 |
| Glucose (mg/dL) | 149.9±70.7 | 139.5±53.3 | 0.001 |
| Platelet count (×10^3^/μL) | 179.2±112.0 | 174.7±119.0 | 0.384 |
| Potassium (mmol/L) | 4.6±1.0 | 4.6±0.8 | 0.627 |
| Sodium (mmol/L) | 138.0±6.3 | 137.1±5.5 | 0.002 |
| GCS | 10.8±3.9 | 10.6±4.6 | 0.344 |
| HR (beats per minute) | 89.6±18.0 | 89.5±17.8 | 0.897 |
| MAP (mmHg) | 76.2±14.4 | 74.6±12.7 | 0.012 |
| RR (breaths per minute) | 21.1±5.5 | 21.0±5.5 | 0.646 |
| SI | 0.8±0.2 | 0.8±0.2 | 0.280 |
| Days of ICU stay before RRT initiation | 2.8±4.2 | 4.7±6.8 | <0.001 |
| Initial RRT modalities, CRRT, % | 466 (29.1%) | 393 (56.6%) | <0.001 |
| Mechanical Ventilation, % | 1,251(78.2%) | 526 (75.8%) | <0.228 |
| Death, % | 548 (34.2%) | 313 (45.1%) | <0.001 |

Abbreviations: CKD, chronic kidney disease; BUN, blood urea nitrogen; FiO_2_, fraction of inspired oxygen; Hgb, hemoglobin; WBC, white blood cell; GCS, Glasgow Coma Scale; HR, heart rate; MAP , mean arterial pressure; RR, respiratory rate; SI, shock index; ICU, intensive care unit; RRT, renal replacement therapy; CRRT, continuous renal replacement therapy.
