# Supplementary material for: Association between predialysis creatinine and mortality in acute kidney injury patients requiring dialysis
Source: PLoS One. 2022 Sep 26;17(9):e0274883. doi: 10.1371/journal.pone.0274883 (PMC9512211; doi:10.1371/journal.pone.0274883)
Supplement: S5 Table — (DOCX) [file pone.0274883.s005.docx]

**Supplement Table 5.** Risk of mortality in patients with high predialysis creatinine levels compared with patients with low predialysis creatinine levels in the MIMIC dataset.

| **Significant variables** | OR (multivariable) |
| --- | --- |
| Creatinine < 4 mg/dL | 1.72 (1.27-2.34, *P* < 0.001) |
| Age | 1.03 (1.02–1.05, *P* < 0.001) |
| Vasopressor | 1.70 (1.03–2.82, *P* = 0.038) |
| BUN | 1.00 (1.00–1.01, *P* = 0.279) |
| FiO2 | 1.01 (1.00–1.02, *P* = 0.045) |
| Anion gap | 1.10 (1.04–1.16, *P* < 0.001) |
| Platelet | 1.00 (0.99–1.00, *P* <0.001) |
| Liver cirrhosis | 3.19 (1.81–5.71, *P* <0.001) |
| GCS | 0.93 (0.88–0.98, *P* =0.005) |
| HR | 0.97 (0.95–1.00, *P* = 0.056) |
| RR | 1.07 (1.03–1.11, *P* = 0.001) |
| Mechanical ventilation | 2.17 (1.27–3.78, *P* = 0.005) |

Abbreviations: BUN, blood urea nitrogen; FiO_2_, fraction of inspired oxygen; GCS, Glasgow Coma Scale; HR, heart rate; RR, respiratory rate.
