# Supplementary figures and images for: Association between predialysis creatinine and mortality in acute kidney injury patients requiring dialysis
Source: PLoS One. 2022 Sep 26;17(9):e0274883. doi: 10.1371/journal.pone.0274883 (PMC9512211; doi:10.1371/journal.pone.0274883)

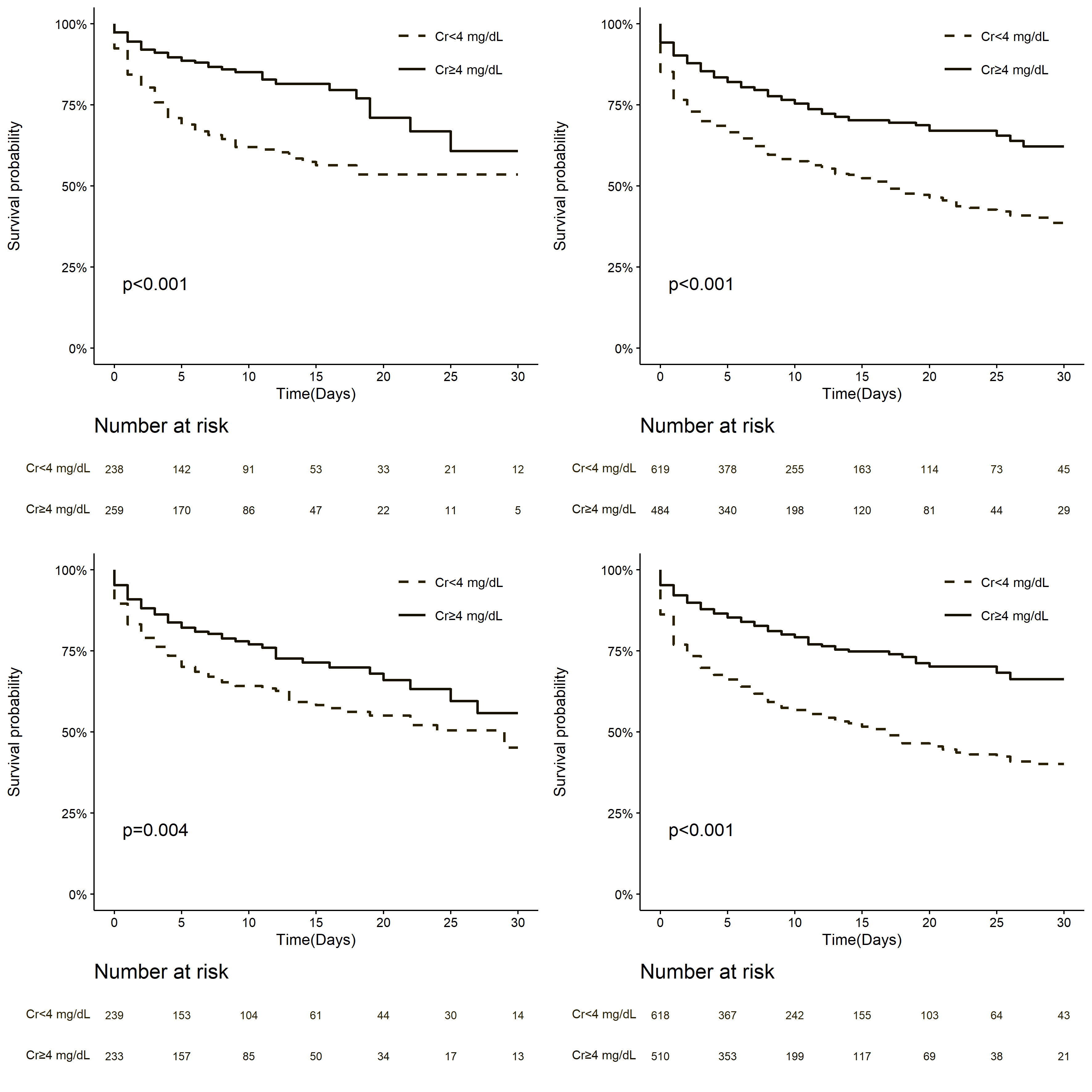

Supplement: S1 Fig — The low-creatinine group had a higher 30-day mortality rate in four groups. (PNG) [file pone.0274883.s006.png]

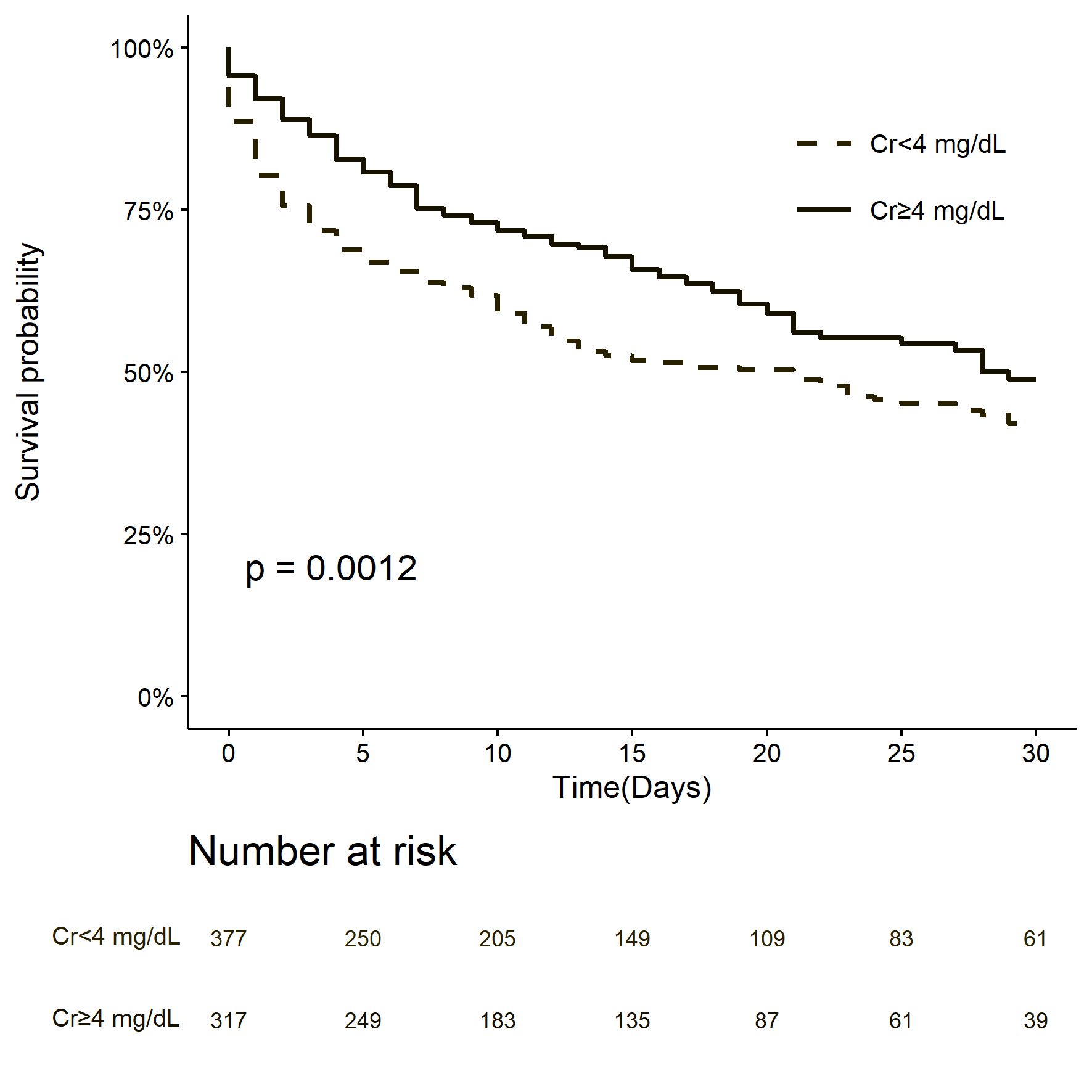

Supplement: S2 Fig — The low-creatinine (Cr < 4 mg/dL) group was associated with worse survival (log rank test, P < 0.0001). (PNG) [file pone.0274883.s007.png]

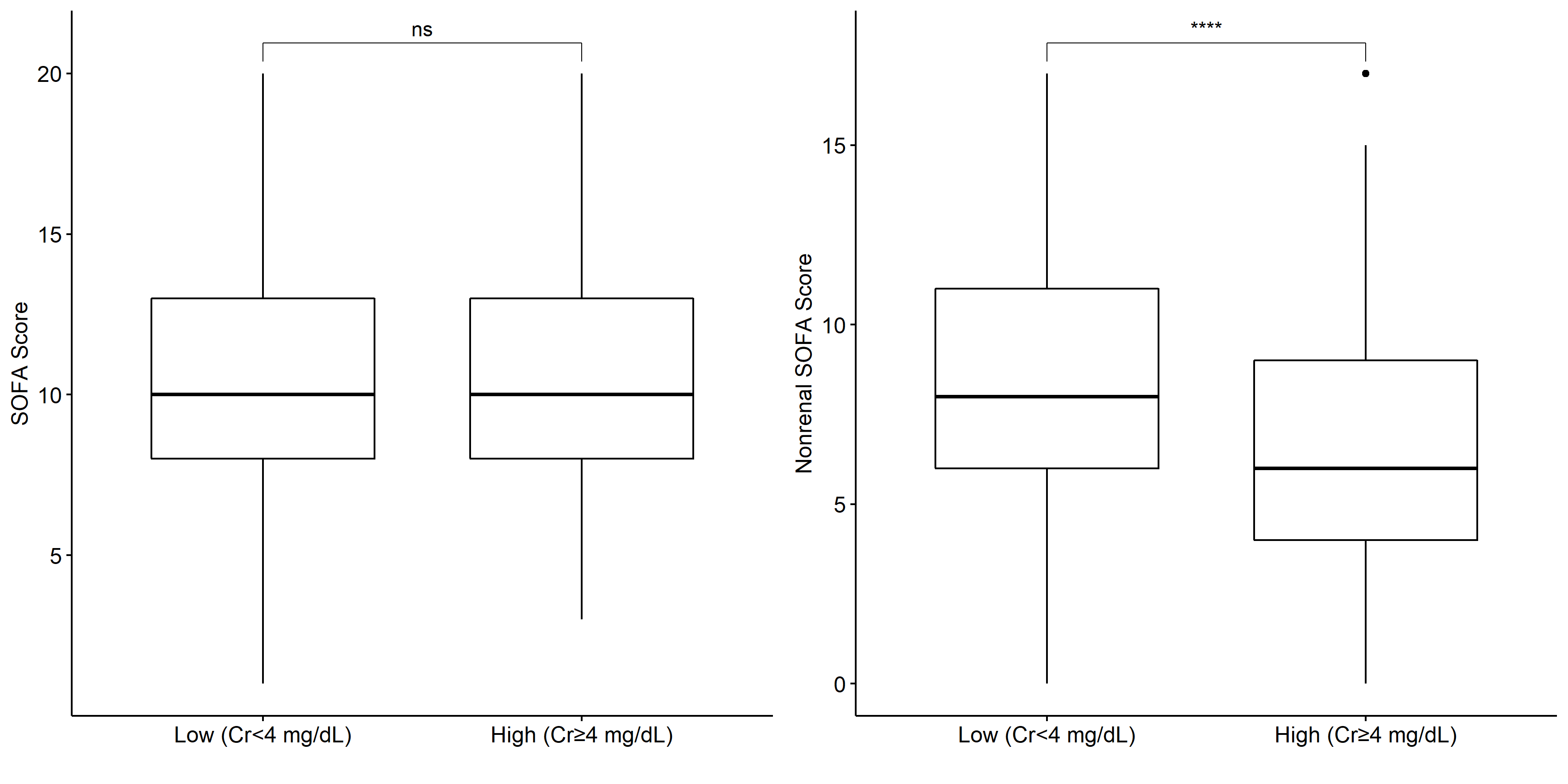

Supplement: S3 Fig — The low-creatinine (Cr < 4 mg/dL) group had a higher nonrenal SOFA score (Kruskal–Wallis test, P < 0.0001). ns: not significant. (PNG) [file pone.0274883.s008.png]

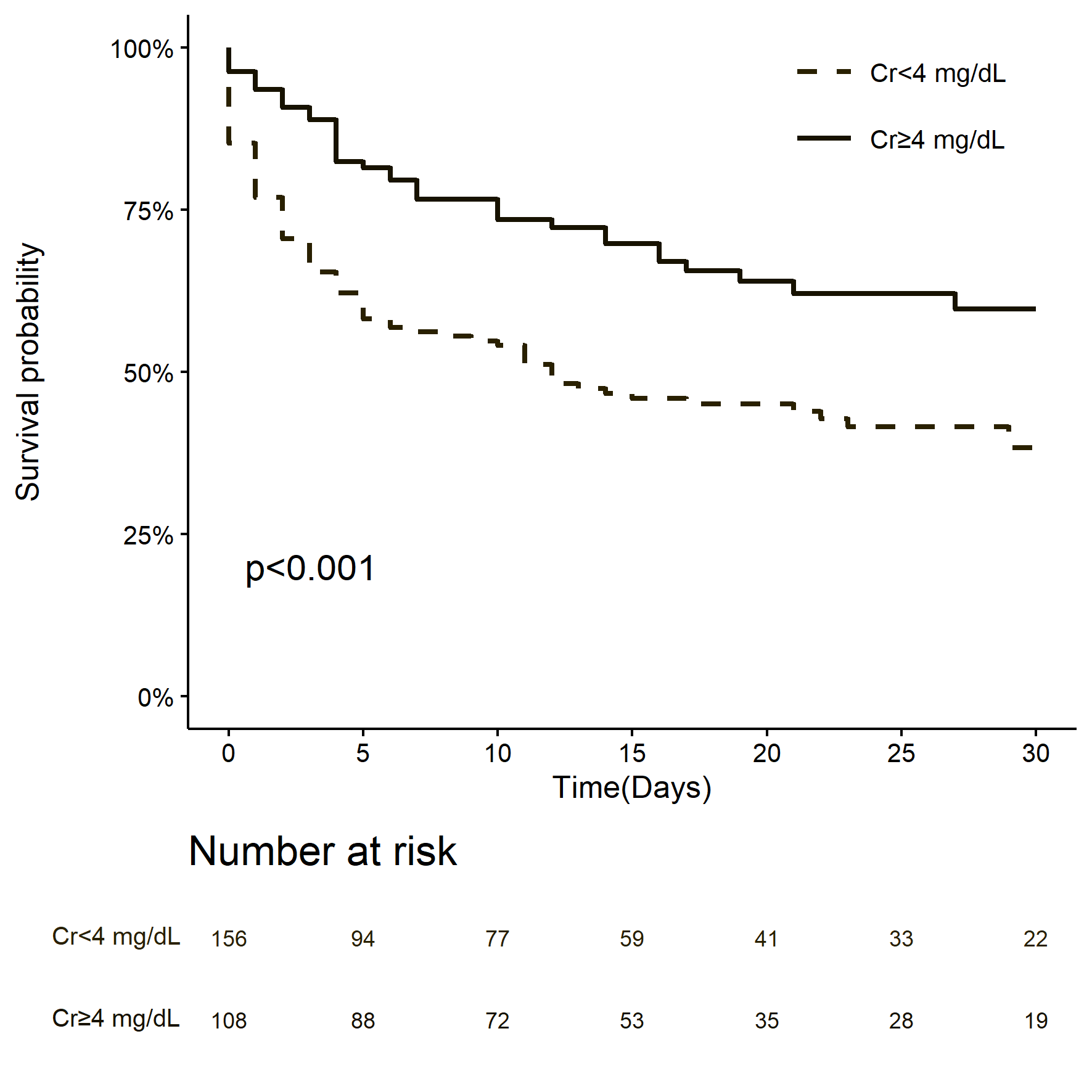

Supplement: S4 Fig — The low-creatinine (Cr < 4 mg/dL) group was associated with worse survival (log rank test, P < 0.001). (PNG) [file pone.0274883.s009.png]

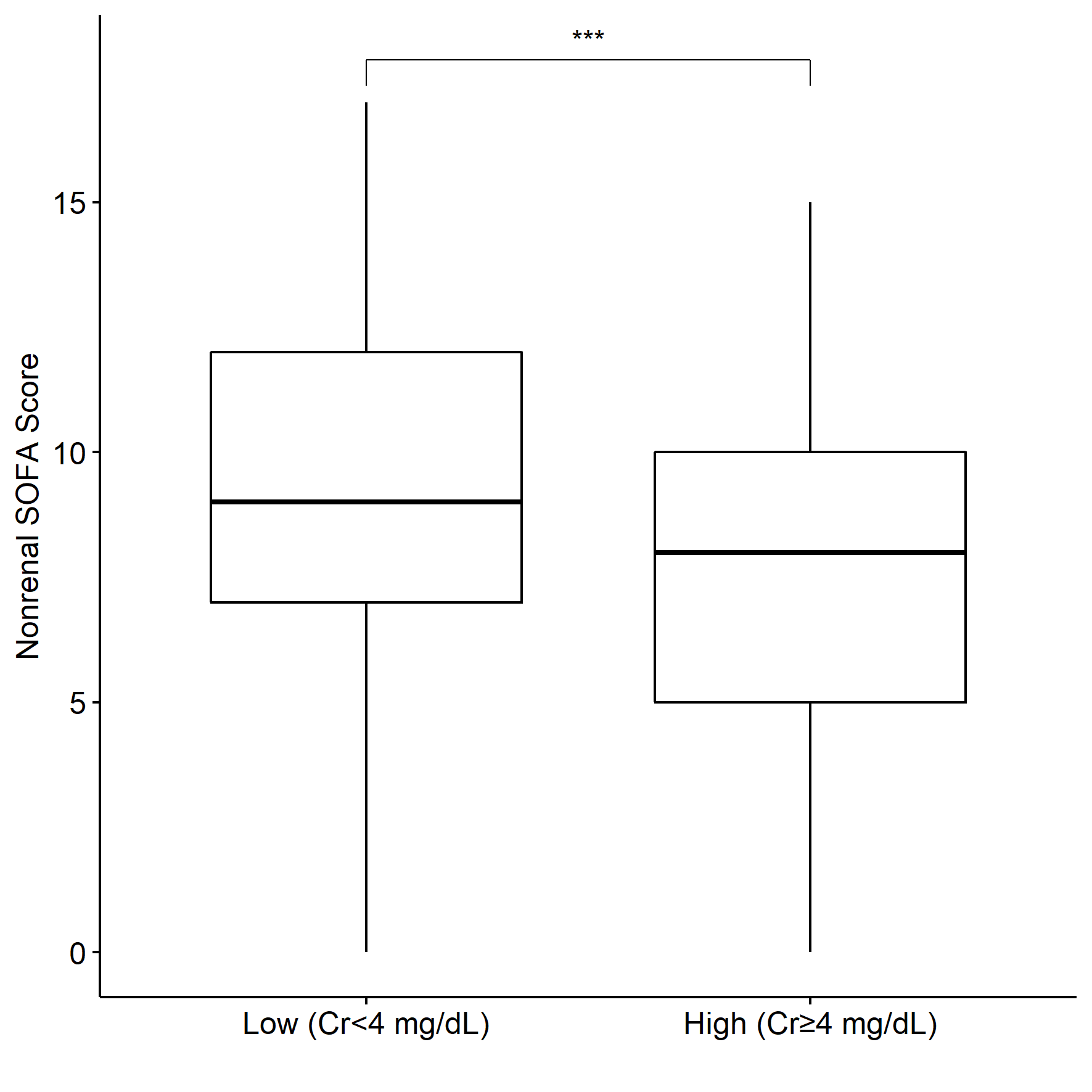

Supplement: S5 Fig — The low-creatinine (Cr < 4 mg/dL) group had a higher nonrenal SOFA score (Kruskal–Wallis test, P < 0.001). (PNG) [file pone.0274883.s010.png]
