# Supplementary material for: Association between predialysis creatinine and mortality in acute kidney injury patients requiring dialysis
Source: PLoS One. 2022 Sep 26;17(9):e0274883. doi: 10.1371/journal.pone.0274883 (PMC9512211; doi:10.1371/journal.pone.0274883)
Supplement: Attachment — Submitted filename: Response to Reviewers.docx [file pone.0274883.s011.docx]

**Response to the Editors’ and Reviewers’ Comments**

We would like to thank the editors and reviewers for the comprehensive assessments, constructive criticisms and valuable comments to our manuscript. Under the recommendations, we have revised our manuscript in response to all comments of two editors and three reviewers. We truly believe that our revised manuscript becomes more clarified with satisfactory changes. The major changes are listed below:

1. The style has been revised to fit all requirements of PLOS ONE guideline (requested by the Editor).

2. We have included our tables as part of our main manuscript and made (requested by the Editor).

3. We removed the reference #10 *Kang MW, Kim J, Kim DK, Oh K-H, Joo KW, Kim YS, et al. Machine learning algorithm to predict mortality in patients undergoing continuous renal replacement therapy. Critical Care. 2020;24(1):42.* because it focused on the prediction of mortality by machine learning.

4. We added the new affiliation to the corresponding author because he got the new offer in the pandemic and had a lot of supports to revised the manuscript in this new hospital.

5. We have clarified most of all questions requested by the reviewer #1.

6. We have clarified all questions requested by the reviewer #2.

**Response to the Editor:**

*Editors' Comments for Author:*

1. *Please ensure that your manuscript meets PLOS ONE's style requirements, including those for file naming.*

**Response:**

We thank the editor for reminding us this issue. We read the guidelines and revised it.

2. *Please include your tables as part of your main manuscript and remove the individual files*

**Response:**

We thank the editor for reminding us this issue. We have revised manuscript and uploaded files as comments.

**Response to Reviewer: 1**

*Comments to the Author*

1. *The authors presented evidence of low creatinine as a risk factor for mortality, if possible to extract from the database more nutritional parameters need to be included i.e BMI .Also if in the table 2 BMI can be included because the low creatinine may reflect a compromised nutritional status (reason for which BMI and other nutritional factors could be included). In the survival analysis (Kaplan Meir curves) in figure 2A analysis by albumin level can be done and may give some answers also BMI can be used if available. Figure2B is it possible to add albumin and BMI and analyze*

**Response:**

We appreciate the reviewer’s critical comments and valuable suggestions. We added albumin and BMI to explore the relationship between creatinine and nutritional status. But we chose to put the results in sensitivity analysis section and put them in supplementary files.

**Response to Reviewer: 2**

*Comments to the Author*

1. *One criticism is that in both the Abstract (Results section) and the Results section itself (page 12), the sentence about the sensitivity analysis which showed that low creatinine patients with fluid overload had worse survival is very poorly and unclearly phrased: "In sensitivity analyses, the low-creatinine group was associated with higher mortality rate in patients as the degree of fluid overload". Please make this phrasing more clear e.g. "In the low-creatinine group, fluid overloaded patients were associated with a significantly worse survival".*

**Response:**

We thank the reviewer for the excellent comment. We made it more clear in the revised manuscript.

2. *In the Results section, I note that the "Predialysis creatinine and SOFA/nonrenal SOFA scores" section on page 11 does not clarify whether there is a statistically significant difference in SOFA/non-renal SOFA scores between the low and high creatinine groups. Please add the relevant P-value to this section, as per Figure 3*.

**Response:**

We thank the reviewer for the comment. We added it in the revised manuscript.
