# Supplementary material for: Association between predialysis creatinine and mortality in acute kidney injury patients requiring dialysis
Source: PLoS One. 2022 Sep 26;17(9):e0274883. doi: 10.1371/journal.pone.0274883 (PMC9512211; doi:10.1371/journal.pone.0274883)
Supplement: Attachment — Submitted filename: Response to Reviewers.docx [file pone.0274883.s012.docx]

**Response to the Editors’ and Reviewers’ Comments**

We would like to thank the editors and reviewers for the comprehensive assessments, constructive criticisms and valuable comments to our manuscript. Under the recommendations, we have revised our manuscript in response to all comments of one editor and two reviewers. We truly believe that our revised manuscript becomes more clarified with satisfactory changes.

**Response to Reviewer:**

1. *suggestion for including the results of BMI in table 1; clarify how the BMI groups were separated to do the analysis of survival; iii) clarify what percentage of patients were fluid overload in each group (low/high creatinine) and include the number in table 1.*

**Response:**

We appreciate the reviewer’s critical comments and valuable suggestions. We added BMI and albumin to explore the relationship between creatinine and nutritional status. But we chose to put the results in sensitivity analysis section and put them in supplementary files (S5 figure) because there are fewer cases if we include BMI and fluid status. We described the percentage of patients with fluid overload in sensitivity analysis section. We didn’t put the data in table1.

2. *Need to mention in page 23 that in the group with a creatinine level >4 there were more male and black race patients that usually have higher baseline creatinine levels; need to mention that the patients with lower creatinine have more days in the ICU before the RRT initiation.*

**Response:**

We thank the reviewer for the excellent comment. We added the comments in the Baseline Characteristics of the Study Cohort section.

3. *Please comment that the creatinine level in these patients may not be the “real” one due to fluid overload and dilution. Did the authors consider to adjust creatinine values to compensate for fluid overload?*

**Response:**

We thank the reviewer for the comment. The fluid status may affect creatinine level. But, a study from Finland recently showed that adjusting Cr values for estimated fluid balance probably has limited value for improving AKI prognostic value for important ICU outcomes (Acta Anaesthesiol Scand 2021 Sep;65(8):1079-1086). We tried to adjust Cr according to Macedo (*Critical Care.* 2010;**14**:R82.) and odds ratio of the high-creatinine group was 0.6 (*P*=0.001). The low-creatinine group still had a higher mortality.

4. *It may be worth adding a sentence to the limitation section about the limitation associated with dichotomizing creatinine as a continuous predictor variable.*

**Response:**

We thank the reviewer for the comment. We added it to the Strengths and Limitations section.
