# Supplementary material for: Association between predialysis creatinine and mortality in acute kidney injury patients requiring dialysis
Source: PLoS One. 2022 Sep 26;17(9):e0274883. doi: 10.1371/journal.pone.0274883 (PMC9512211; doi:10.1371/journal.pone.0274883)
Supplement: Attachment — Submitted filename: Response to Reviewers_08222022.docx [file pone.0274883.s013.docx]

**Response to the Editors’ and Reviewers’ Comments**

We would like to thank the editors and reviewers for the comprehensive assessments, constructive criticisms and valuable comments to our manuscript. Under the recommendations, we have revised our manuscript in response to all comments of one editor and two reviewers. We truly believe that our revised manuscript becomes more clarified with satisfactory changes. The major changes are listed below:

1. We appreciate the valuable comments. We revised the manuscript as comments. We included BMI and fluid overload variables in our study. There were 1600 and 694 patients in the eICU and MIMIC, respectively. We revised all the tables and figures accoring to the new study group and also the statitistic results.
